# Defect and Particle-Size Engineering as Mechanistic Drivers for Dye Uptake in a Zirconium Metal–Organic Framework

**DOI:** 10.1021/acsomega.6c00601

**Published:** 2026-06-09

**Authors:** Karl Thomas Jackson, Robert H. Lomax, Fatemeh Parnianchi

**Affiliations:** Department of Chemistry, College of Natural and Health Sciences, 6522 Virginia State University, Petersburg ,Virginia 23806,United States

## Abstract

Precise control over the particle size and defect density of metal–organic frameworks (MOFs) is critical for optimizing their performance in adsorption-based applications. Acid modulation offers a powerful route for tuning structural features in MOFs enabling control over adsorbate–adsorbent interactions. In this study, UiO-66 was synthesized using 75, 150, and 300 equiv of acetic acid relative to zirconium (designated as Z01, Z02, and Z03, respectively) to investigate how defect density and particle size influence dye adsorption mechanisms. Increasing acid concentration produced a direct increase in particle size (190, 330, and 450 nm, respectively) and an inverse trend in dye adsorption capacity across all three probe molecules—methyl orange, methylene blue, and curcumin. Surface area (950–1160 m^2^/g) and defect levels (0.6–1.1) exhibited a nonlinear dependence on the acid concentration, increasing from 75 to 150 equiv before decreasing at 300 equiv kinetic analysis showing dye- and sample-dependent behavior, with PSO providing the best overall description for MO and MB adsorption, while curcumin exhibited mixed kinetic behavior. Isotherm modeling of methyl orange and methylene blue on Z01 showed nonlinearity in Langmuir fits but strong linearity in Freundlich plots, consistent with heterogeneous surface adsorption. Across all materials, methyl orange exhibited the highest uptake and methylene blue the lowest. Variation of reaction time (6–48 h) had no measurable effect on structural properties or adsorption performance. These results demonstrate that acid-modulated defect and particle-size engineering provide a mechanistic handle for tuning dye adsorption behavior in MOFs, offering a pathway toward rational design of adsorbents with tailored interaction profiles.

## Graphical abstract

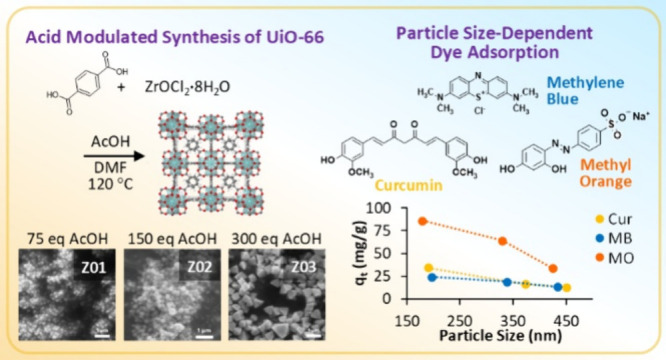

## 1 Introduction

The removal of synthetic dyes from aqueous environments is a critical environmental challenge due to their persistence, toxicity, and widespread use in textile, pharmaceutical, and food industries.^1–3^ Over 100,000 commercially available dyes are used worldwide, with an estimated 700,000 tons produced annually.^4,5^ A significant fraction (10–15%) of these dyes is discharged into wastewater during manufacturing and processing, often without complete treatment.^6^ Even small concentrations (less than 1 mg/L) of certain dyes can block sunlight penetration in water, disrupting photosynthesis and aquatic ecosystems.^7^

Beyond ecological damage, numerous dyes and their degradation products are associated with serious health risks. Azo dyes, for example, can undergo reductive cleavage to form aromatic amines, many of which are mutagenic and carcinogenic.^8,9^ Exposure to dye-contaminated water has been linked to skin irritation, respiratory issues, endocrine disruption, and increased cancer risk in humans.^9^ These combined environmental and health concerns underscore the urgent need for efficient, selective, and robust technologies for dye removal from water systems.

Addressing this challenge requires advanced materials capable of selective and efficient dye removal. Metal–organic frameworks (MOFs) are a class of porous crystalline materials particularly suited for this task because of their high surface area, tunable pore structure, and customizable chemical functionality.^10–12^ Among these, UiO-66 (University of Oslo-66) has emerged as a benchmark adsorbent for aqueous applications. Its zirconium-based nodes (Zr_6_O_4_(OH)_4_) form exceptionally strong coordination bonds with terephthalate linkers, imparting remarkable thermal, chemical, and hydrolytic stability—even under harsh aqueous and acidic/basic conditions where many other MOFs degrade.^13,14^ In addition to its robustness, UiO-66 is uniquely suited for studying adsorption mechanisms because of its high tolerance for structural defects. Crystal defects can be deliberately introduced during synthesis without collapsing the framework, generating altered pore environments that enhance adsorption affinity and accessibility.^14,15^ Yet despite extensive study, the mechanistic origins of adsorption behavior in UiO-66 remain incompletely understood, particularly with respect to the combined influence of particle size and defect density on mass-transfer pathways.

Two structural parameters exert especially strong influence on adsorption: defect density and particle size. Missing-linker defects introduce open metal sites and modify pore environments, often enhancing adsorbate interactions and internal surface accessibility.^14,15^ Particle size, in contrast, dictates external surface area, diffusion length, and boundary-layer resistance.^16,17^ While both parameters are known to affect adsorption, they are often varied simultaneously, making it difficult to isolate their individual contributions, leaving questions about their relative contributions to adsorption kinetics, equilibrium behavior, and mechanistic pathways.

Acid modulation provides a powerful synthetic handle for tuning defect formation and crystal growth in UiO-66.^14,15,18^ Marshall et al. published a comprehensive analysis of strategies for controlling MOF particle size.^19^ The study proposes that modulation of particle size depends on the competition between four chemical equilibria: linker deprotonation, modulator deprotonation, linker complexation, and termination. This framework gives rise to a characteristic “see-saw” relationship, wherein increasing acidic ligand concentration initially decreases particle size via surface capping, followed by size growth beyond a threshold as metal-ion diffusion outpaces termination. While such studies provide critical insight into synthetic control of MOF structure, the mechanistic consequences of this structural tuning—particularly how particle size and defect density together influence adsorption pathways—remain largely unexplored.^20,21^ Much of the prior work emphasizes equilibrium uptake or surface-area correlations, without explicitly linking structural evolution to kinetic models, intraparticle diffusion behavior, or transport limitations. Dye adsorption studies on UiO-66 and related Zr-MOFs further demonstrate that molecular properties such as charge, planarity, and steric bulk strongly influence uptake behavior.^20–22^ However, these studies typically assess adsorption performance without connecting dye-specific behavior to deliberately engineered framework features such as defect density or particle size. As a result, further exploration is needed for a mechanistic understanding of how structural tuning modulates dye-dependent transport and adsorption pathways.

In this work, we address these gaps by synthesizing UiO-66 under controlled acetic-acid modulation to generate materials with systematically varied particle sizes and defect densities. Using three structurally distinct dyes—methyl orange (anionic), methylene blue (cationic), and curcumin (neutral)—we probe how engineered structural features govern adsorption mechanisms across diverse molecular profiles (Figure 1). Unlike prior studies that primarily treat structural parameters independently, this work deliberately decouples particle size and defect density to isolate their individual and combined effects on adsorption behavior. By integrating kinetic modeling (PFO, PSO, IPD), isotherm analysis (Langmuir, Freundlich), and detailed structural characterization, we demonstrate that particle size and defect architecture function as mechanistic levers that control dye uptake pathways rather than merely equilibrium capacity. The novelty of this work lies in positioning particle size and defect density not merely as synthetic outcomes, but as tunable mechanistic variables that govern dye-dependent adsorption pathways, providing a more comprehensive framework for understanding adsorption in MOFs and informing the rational design of porous materials for selective removal of organic contaminants from aqueous environments.

**Figure 1:**
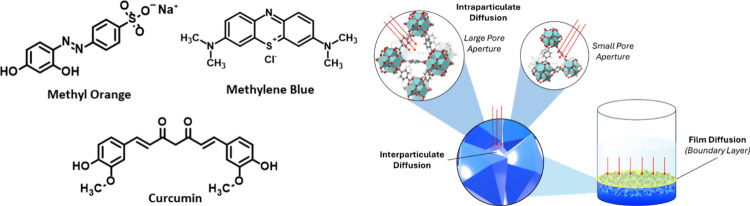
Chemical structures of methyl orange, methylene blue, and curcumin (left) alongside schematic representations of dye transport mechanisms in porous materials (right).

## 2 Materials and Methods

### 2.1 Chemicals

Zirconyl chloride octahydrate (ZrOCl_2_·8H_2_O), 1,4-benzenedicarboxylic acid (H_2_BDC), *N*,*N*-dimethylformamide (DMF, anhydrous), acetic acid (glacial), methanol, methyl orange (MO), methylene blue (MB), and curcumin (CUR) were purchased from Fisher Scientific and used as received without further purification.

### 2.2 Synthesis of UiO-66

UiO-66 nanoparticles of varying sizes and defect densities were synthesized by a modulated solvothermal method based on established UiO-66 synthesis and defect-engineering strategies.^13,15,23,24^ In a typical procedure, 50 mg (0.30 mmol) of H_2_BDC was dissolved in 10 mL of DMF along with varying amounts of glacial acetic acid (75, 150, or 300 eqs relative to Zr) in a 100 mL Duran bottle under magnetic stirring. Separately, 70 mg (0.30 mmol) of ZrOCl_2_·8H_2_O was dissolved in the appropriate volume of DMF by sonication. The volume of DMF and AcOH for all experiments totaled 25 mL. The zirconium solution was added to the Duran bottle, which was then sealed and placed in an oven at 120 °C for varying reaction times (6–48 h). The resulting white precipitate was separated from the mother liquor by centrifugation (10,000 rpm, 10 min) and soaked in fresh DMF overnight. The product was subsequently washed three times with 15–20 mL portions of methanol, with centrifugation between each wash. The final solid was dried at 70 °C for several hours prior to characterization. Samples prepared at reaction times of 6 h using 75, 150, and 300 eq of AcOH were designated Z01, Z02, and Z03, respectively, and will be the primary focus of this study.

### 2.3 Characterizations

Powder X-ray diffraction (PXRD) patterns were collected using a Bruker D2 Phaser diffractometer with Cu Kα radiation (λ = 1.5418 Å) to confirm crystallinity and phase purity. Nitrogen adsorption–desorption isotherms were measured at 77 K using a Quantachrome NovaTouch LX2 surface area analyzer. Prior to analysis, samples were degassed under vacuum at 120 °C for 12 h. Brunauer–Emmett–Teller (BET) surface areas and pore volumes were calculated from the adsorption branch.

Fourier-transform infrared (FT-IR) spectra were collected on a Shimadzu IRSpirit spectrometer using the attenuated total reflectance (ATR) attachment. Thermogravimetric analysis (TGA) was carried out under air flow using a TA Instruments TGA-55 analyzer from room temperature to 600 °C. The heating rate began at 10 °C/min until 300 °C, then slowed to 5 °C/min until 400 °C, after which the rate was increased back to 10 °C/min until completion. UV–visible spectra for adsorption studies were measured using a ThermoFisher GENESYS 150 UV–vis spectrophotometer. Scanning electron microscopy (SEM) images were obtained using a JEOL JSM-7200F field-emission microscope. Samples were prepared by drop-casting dilute methanolic suspensions onto a carbon substrate and dried under ambient conditions without conductive coating. Particle size distributions were determined from SEM micrographs using ImageJ software, with at least ten particles measured per image across three independently prepared samples, providing consistent relative size trends across samples. Dynamic light scattering (DLS) measurements were conducted using an Anton Paar Litesizer DLS 700 to corroborate particle size trends. Measurements were performed in dilute methanolic or ethanolic suspensions using side-scatter detection (90°). Intensity-weighted size distributions were used for comparison of relative size trends rather than absolute hydrodynamic diameters. Only measurements with transmittance ≥70% and polydispersity index (PDI) ≤25% were included.

### 2.4 Batch Adsorption Experiments

Batch adsorption experiments were conducted to assess the uptake of MO, MB, and Cur. In each trial, 8 mg of UiO-66 sample was added to 4 mL of 500 mg/L dye solution. MO and MB were both aqueous solutions while Cur was dissolved in ethanol due to its limited aqueous solubility. After 3 h, solids were separated by centrifugation, and the residual dye concentration in the supernatant was determined by UV–vis spectroscopy at a λ_max_ = 425, 465, and 664 nm for Cur, MO, and MB, respectively. Adsorption capacity was calculated based on a calibration curve. Adsorption capacity, *q_t_* (mg/g) was determined using the following equation:

$$q_{t}=V\frac{\left(C_{0}-C_{t}\right)}{m}$$ 1

where *C*_0_ and *C_t_* (mg/L) are the initial and time-dependent dye concentrations, *V* (L) is the solution volume, and *m* (g) is the mass of adsorbent.

To gain insight into adsorption mechanisms, isotherm analyses were conducted using the linearized Langmuir and Freundlich models where initial dye concentrations ranged from 10 to 500 mg/L. Adsorption capacities (*q_t_*) for these models were determined after 90 min. The Langmuir model assumes monolayer adsorption onto a homogeneous surface with a finite number of identical sites and is expressed as

$$\frac{C_{e}}{q_{e}}=\frac{1}{q_{\max}K_{L}}+\frac{C_{e}}{q_{\max}}$$ 2

where *C*_e_ (mg/L) is the equilibrium dye concentration, *q*_e_ (mg/g) is the adsorption capacity at equilibrium, *q*_max_ (mg/g) represents the maximum monolayer adsorption capacity, and *K*_L_ (L/mg) is the Langmuir adsorption constant. The Freundlich model, which accounts for surface heterogeneity and nonuniformity in adsorption energies, is described by equation:

$$\ln\;q_{e}=\ln\;K_{F}+\frac{1}{n_{F}}\ln\;C_{e}$$ 3

where *K*_F_ and *n* are the Freundlich constants related to adsorption capacity and adsorption intensity, respectively. All adsorption experiments were performed in triplicate, and the reported values represent the mean ± standard deviation.

### 2.5 Adsorption Kinetics Study

Adsorption kinetics experiments were carried out using initial dye concentrations of 15 mg/L for Cur and MO, and 10 mg/L for MB. Absorbance measurements were recorded at 15 min intervals over a total duration of 180 min, and adsorptive capacities were determined as described above. To investigate the adsorption kinetics and possible rate-limiting mechanisms, time-dependent adsorption data were evaluated using pseudo-first-order (PFO) and pseudo-second-order (PSO) kinetic models. The linearized form of the PFO model is given by

$$\ln\left(q_{e}-q_{t}\right)=\ln\;q_{e}-k_{1}t$$ 4

where *q*_e_ (mg/g) and *q_t_* (mg/g) are the adsorption capacities at equilibrium and time, *t*, respectively, and *k*_1_ (min^–1^) is the PFO rate constant. The PSO kinetic model is expressed as

$$\frac{t}{q_{t}}=\frac{1}{k_{2}q_{e}^{2}}+\frac{t}{q_{e}}$$ 5

Because all experiments were conducted under identical quiescent conditions, the extracted kinetic parameters are interpreted as comparative indicators of adsorption behavior rather than intrinsic surface-reaction rate constants.

To further probe mass-transfer contributions, the intraparticle diffusion (IPD) model was applied. This model accounts for the influence of boundary-layer diffusion and can provide insight into whether intraparticle diffusion is the predominant rate-determining step. The IPD model is expressed as

$$q_{t}=k_{\mathrm{id}}t^{1/2}+C$$ 6

where *q_t_* (mg/g) is the adsorption capacity at time *t*, *k*_id_ (mg/g·min^1/^ ^2^) is the intraparticle diffusion rate constant, and *C* (mg/g) is a constant related to the thickness of the boundary layer. Larger values of *C* indicate a greater contribution from external mass-transfer resistance.

#### 2.5.1 Solution Conditions

All adsorption experiments (MO, MB) were performed using deionized water without buffer or added salts; curcumin solutions were prepared in ethanol due to limited aqueous solubility. No pH or ionic-strength adjustments were made. Experiments were conducted under quiescent (nonagitated) conditions. The absence of external mixing enhances sensitivity to differences in boundary-layer resistance and pore accessibility, enabling clearer comparative evaluation of the three MOF samples under identical conditions. Reported adsorption capacities and kinetics should be interpreted with these constraints in mind.

## 3 Results and Discussion

Extant adsorption studies have demonstrated that UiO-66 and related Zr-MOFs show strongly adsorbate-dependent dye uptake. Acid-promoted UiO-66 has been reported to display more regular octahedral morphology and selective adsorption behavior toward anionic dyes such as methyl orange in the presence of cationic dyes such as methylene blue.^25^ Other work has examined methylene blue adsorption on UiO-66 as a function of solution conditions and found that uptake depends strongly on pH, dose, and contact time.^26^ Mesoporous UiO-66 has likewise been shown to enhance dye mass transfer in MO- and MB-containing systems,^27^ and UiO-66-based sorbents have also been investigated for curcumin adsorption.^28^ Importantly, defect-controlled UiO-66 has already been examined for MO and MB removal, indicating that defect generation can increase active adsorption sites and alter adsorption capacity and selectivity.^29^

These findings underscore the importance of understanding how synthetic variables translate into mechanistic differences during dye uptake. To probe these effects in a controlled manner, we focus our analysis on UiO-66 samples prepared at 75, 150, and 300 equiv of acetic acid (Z01, Z02, and Z03) and at various reaction times to explore their impact on particle size and defect level. Because variation in reaction time from 6 to 48 h produced no measurable differences in structural characterizations or adsorption performance, the discussion that follows focuses primarily on 6-h experiments, unless otherwise indicated.

### 3.1 Structural Characterization

To evaluate how modulator concentration influences the structural and morphological features of UiO-66, we first examined the crystallinity and phase purity of the three materials using PXRD and FT-IR. PXRD patterns were collected for Z01, Z02, and Z03 and compared to the simulated UiO-66 structure. Samples prepared under varying reaction times and acetic acid eqs exhibited the expected crystalline structure with no observable differences in their diffraction patterns within the resolution of PXRD, which is primarily sensitive to long-range crystallinity. (Figure 2A). The PXRD patterns of all MOFs showed characteristic peaks consistent with UiO-66 at 2θ = 7.4°, 8.6°, and 25.7° representing reflections (111), (002), and (305), respectively, among other prominent peaks matching the simulated pattern of UiO-66.^13,30^

**Figure 2:**
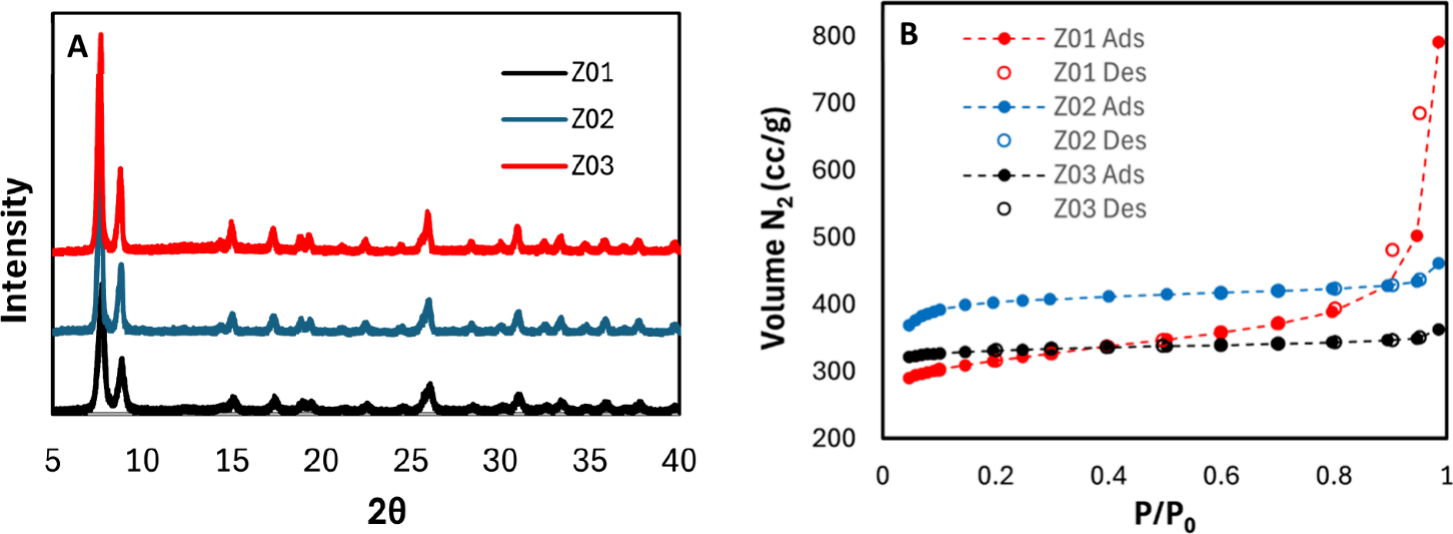
(A) Powder X-ray diffraction (PXRD) patterns confirming crystallinity and phase purity of the MOF samples. (B) Nitrogen sorption isotherms for UiO-66 samples synthesized with varying acetic acid concentrations.

FT-IR was used to assess coordination of the 1,4-benzenedicarboxylate (BDC) linker in samples Z01–Z03 (Figure S5, Supporting Information). The three spectra are highly similar, indicating that all samples possess the same local bonding environment characteristic of UiO-66. Bands at ca. 1580 cm^–1^ and 1390 cm^–1^ are assigned to the asymmetric and symmetric stretching vibrations of coordinated carboxylate groups, ν_as_(COO−) and ν_s_(COO−), respectively.^31^ The feature near 1505 cm^–1^ is attributed to C=C aromatic ring skeletal vibration of the BDC linker and may partially overlap with ν_as_(COO−) at 1580 cm^–1^,^32^ while the peak around 1015 cm^–1^ can be attributed to the C–H bending vibration.^33^ Additional absorptions in the ca. 750–650 cm^–1^ region are consistent with the Zr–O and Zr–O–C stretching vibrations associated with the Zr_6_O_4_(OH)_4_ metal cluster and may overlap with aromatic C–H out-of-plane bending. Notably, no strong band attributable to the C=O stretching vibration of free H_2_BDC is observed near ∼1700 cm^–1^,^32^ supporting linker deprotonation and coordination to the Zr nodes. Overall, the FT-IR data are consistent with formation of the UiO-66 framework in all three samples.

### 3.2 Nitrogen Sorption Data

Nitrogen sorption isotherms were collected for samples Z01, Z02, and Z03 to evaluate surface area and porosity. Z02 and Z03 exhibit Type I isotherms, characteristic of microprous materials, while Z01 exhibited Type IV isotherms, indicative of mesoporous or mixed mesoprous/microporous materials (Figure 2B).^34^ Key observable differences include a sharp uptake in adsorption at higher relative pressures (*P*/*P*_0_ ≥ 0.9 as well as a slight hysteresis in the same range) for Z01. This behavior suggests the presence of a small fraction of secondary porosity beyond the intrinsic micropore network.^35^ The steep uptake near saturation pressure is commonly attributed to either defect-associated mesoporosity or interparticle voids arising from crystallite packing.^36^ A defect-origin for this feature is unlikely as Z02, despite exhibiting a higher defect density, does not display comparable high-pressure uptake or hysteresis. The most plausible origin of this behavior is capillary condensation within confined interparticle voids. The accompanying hysteresis, though modest, further supports this, consistent with a narrow distribution of mesopores or structural irregularities not captured by the primary microporous framework. In contrast, the larger particles produced at higher acetic acid eqs (Z02 and Z03) form more open interparticle domains that behave effectively as macroporous voids, which do not support capillary condensation near saturation pressure and therefore do not exhibit comparable hysteresis or high-pressure uptake.

BET surface areas were calculated from the linear region of the isotherms and found to be 1048 ± 54.3 m^2^/g for Z01 (75 eq), 1126 ± 45.2 m^2^/g for Z02 (150 eq), and 944 ± 72.2 m^2^/g for Z03 (300 eq). Surface area increases from 75 to 150 eqs, then decreases at 300 eqs, despite continued particle growth. The decrease in BET surface area at 300 eqs of acetic acid is attributed to enhanced crystallinity and reduced defect concentration rather than framework degradation. Excess modulator slows nucleation and promotes linker equilibration, leading to larger, more ordered UiO-66 crystals with surface areas closer to those of ideal, defect-poor frameworks.

### 3.3 Particle Size Analysis

SEM provided insight into particle morphology and size evolution across the series, enabling direct correlation between AcOH concentration and the resulting particle dimensions and shape. Particle sizes were determined directly from ImageJ measurements of the SEM micrographs. For each micrograph, at least ten particles were measured and averaged, and this procedure was repeated across three replicate samples to ensure statistical reliability. No measurable correlation between particle size and reaction time was observed, indicating that particle growth reaches equilibrium after approximately 6 h (see Supporting Information). Particle size increased systematically with acetic acid concentration, with average diameters of 185 ± 16.9 nm, 301 ± 43.9 nm, and 467 ± 128.1 nm for 75, 150, and 300 eqs, respectively (Figure 3). Morphology also evolved with increasing modulator concentration, transitioning from more spherical at low acid eqs to increasingly well-defined octahedrons at higher eqs.

**Figure 3:**
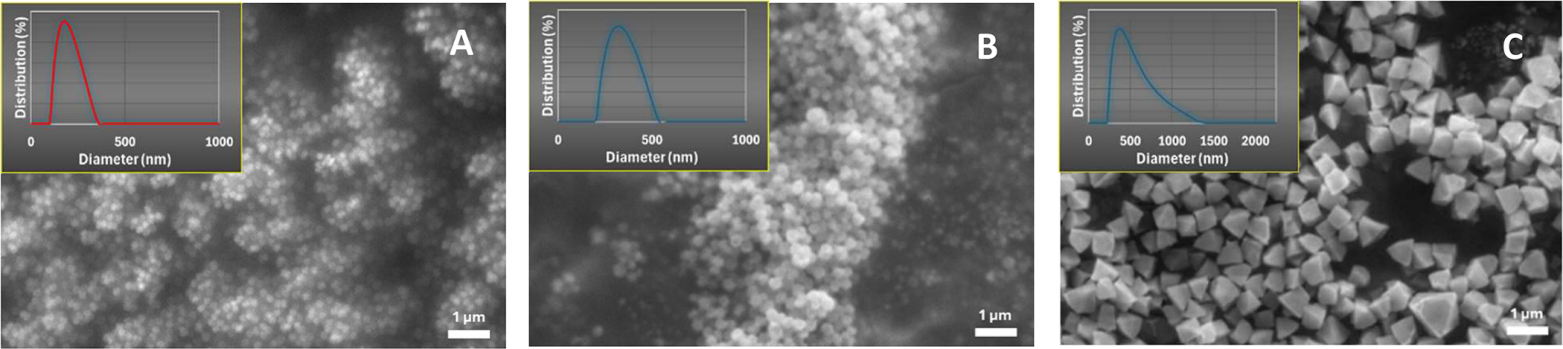
Scanning electron micrographs of samples (A) Z01, (B) Z02, and (C) Z03. Insets show particle size distributions as determined by DLS.

To confirm particle size, DLS was used. For the sake of comparison, peak intensity from size distribution data was used instead of the hydrodynamic diameters. All analyses were conducted using side-scatter detection (90°). Measurements where transmittance was 70% or greater with a polydispersity index (PDI) below 25% were included in the analysis data. Results matched very closely with particle size data from SEM. Averaged across reaction times displayed the same monotonic trend while increasing modulator concentration yielded progressively larger particles (Figure 3 insets). DLS analysis exhibited average diameters of 190 ± 26.9 nm, 364 ± 34.4 nm, and 455 ± 100.1 nm for samples prepared with 75, 150, and 300 eqs of AcOH, respectively. At 75 eqs, both DLS and SEM indicated relatively narrow size distributions, consistent with the formation of small, uniform particles at low modulator concentration. A comparison of particle size from DLS vs SEM can found in Table 1 along with the surface area and defect level of each sample.

**Table 1:** Comparison of Particle Size, Surface Area, and Defect Density of Z01, Z02, and Z03

	**particle size** (nm)				
**MOF**	SEM	**DLS**	solvent	surface area (m^2^/g)	defect level (missing linker)
Z01	184 ± 16.9	190 ± 26.9	ethanol	1048 ± 54.3	0.90 ± 0.10
Z02	301 ± 43.9	364 ± 34.4	methanol	1126 ± 45.2	1.07 ± 0.05
Z03	467 ± 128	455 ± 100	methanol	944 ± 72.2	0.73 ± 0.03

### 3.4 Defect Density

Defect levels in the UiO-66 samples were quantified using a TGA consistent with established literature approaches for missing-linker analysis in Zr-based MOFs (Figure 4). The final mass obtained at the end of the TGA run was assigned to the inorganic ZrO_2_ residue and normalized to 100%. The mass at 390 °C was then expressed as a percentage of this final residue (Wt%@390). For a defect-free UiO-66 framework, the dehydroxylated composition Zr_6_O_6_(BDC)_6_ corresponds to a theoretical mass of 220% relative to ZrO_2_, as previously reported by Driscoll et al. and Athar et al.^37,38^ For each sample, the organic mass fraction present at 390 °C was calculated as Wt%@390, and the fraction of occupied linker sites, *f*, was determined using

$$f=\frac{\mathrm{Wt}\%@390-100\%}{220\%-100\%}$$

multiplying this ratio by six yields the number of linkers per Zr_6_ node, and the defect value, *x*, was defined as

x=6−linkersperformulaunit

**Figure 4:**
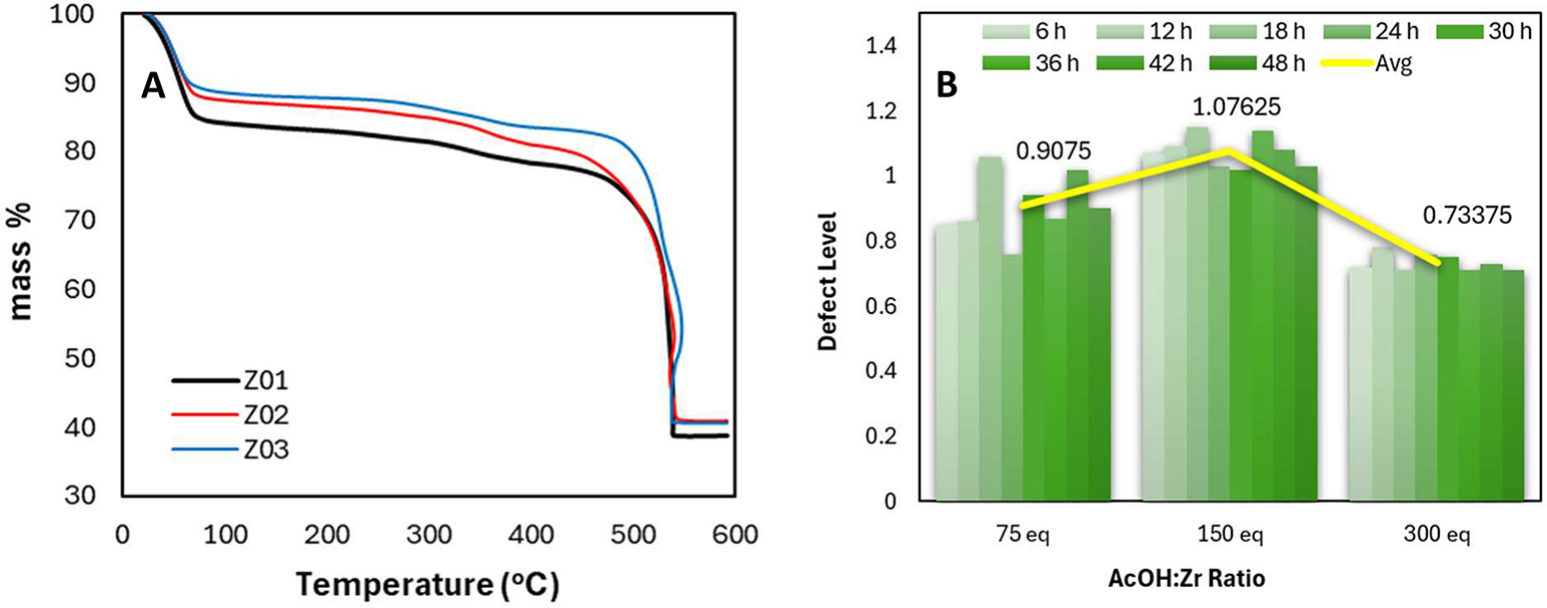
Characterization of UiO-66 samples: (A) Thermogravimetric analysis (TGA) curves showing thermal stability and framework decomposition profiles. (B) Defect levels for materials synthesized at 75, 150, and 300 equiv, shown across reaction times from 6 to 48 h.

Across the series, the samples exhibited defect values ranging from approximately 0.7–1.3, indicating a moderately defective UiO-66 framework. These values are consistent with the defect ranges typically observed for modulated syntheses of UiO-66.

Analysis of the defect levels revealed clear differences among the three acetic acid concentrations. As shown in Figure 4, the 150 eq series exhibited the highest defect density (*x* = 1.07 ± 0.05), followed by the 75 eq samples (*x* = 0.90 ± 0.10), while the 300 eq series consistently showed the lowest defect levels (*x* = 0.73 ± 0.03). These trends directly correlate with the measured BET surface areas. The parallel ordering of defect density and surface area confirms that missing-linker defects significantly enhance porosity and accessible surface area in UiO-66. The low standard deviations observed for the 150 eq and 300 eq series further indicate that defect formation under these conditions is highly reproducible.

### 3.5 Batch Adsorption Behavior

We examined the adsorption behavior of UiO-66 toward MO, MB, and Cur to investigave relative dependence on surface area, defect level, particle size, and dye molecular characteristics. Z01 exhibited the highest adsoprtion capacity of for all three adsorbates followed by Z02, then Z03 Although the 150-equivalent series (Z02) exhibits the highest defect density and BET surface area, the 75-equivalent series (Z01) consistently achieves the greatest adsorption capacity under identical conditions (Figure 5 and Table 2). This outcome is attributed to Z01’s significantly smaller particle size, which increases external accessibility to adsorption sites.^39^ In contrast, Z02 shows intermediate capacities because its larger particles offset the benefits of higher internal porosity, while Z03 exhibits the lowest capacities, consistent with its low defect density, reduced surface area, and largest particle size.

**Figure 5:**
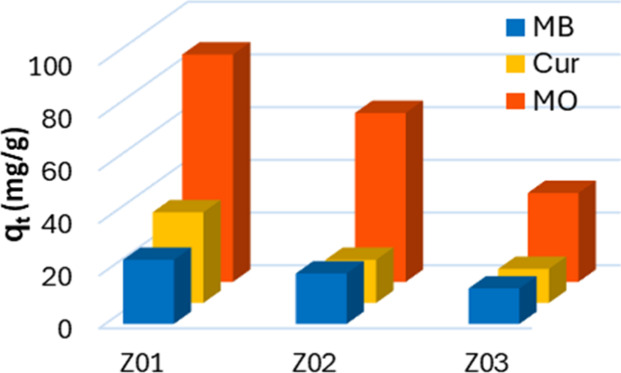
Comparative adsorption capacities of Zr-based MOF samples (Z01, Z02, and Z03) toward methylene blue (MB), curcumin (Cur), and methyl orange (MO).

**Table 2:** Dye Adsorption Capacity, *q*_*t*_, for Z01, Z02, and Z03 (mg/g)

MOF	MB	Cur	MO
Z01	24.3 ± 5.7	37.4 ± 8.7	86.3 ± 29.5
Z02	13.9 ± 2.1	16.4 ± 3.9	64.1 ± 10.4
Z03	11.6 ± 0.7	12.9 ± 2.9	33.7 ± 10.8

Under the tested conditions, the samples with smaller particle sizes consistently exhibit higher adsorption capacities across all three dyes (Figure 6). Although defect creation increases internal surface area, particle size exerts a stronger influence on equilibrium uptake than defect density within the parameter range studied.^35,40^ Dye-specific behavior further reflects the interplay of charge and steric effects: MO demonstrates the strongest adsorption due to electrostatic attraction between its anionic sulfonate group and the slightly positive UiO-66 surface near neutral pH, combined with its planar geometry that facilitates pore access. Curcumin, being sterically bulky and less polar overall, penetrates the microporous interior poorly and therefore depends more heavily on small particle size than on defect density.^41,42^ MB follows a similar accessibility-limited pattern, with uptake highest for Z01 and lowest for Z03.

**Figure 6:**
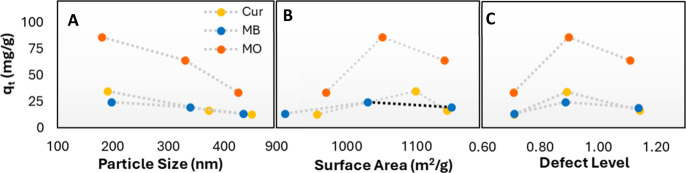
Correlation of adsorption capacity, *q_t_* (mg/g), with key material properties across all UiO-66 samples including (A) particle size (nm), (B) defect level, and (C) surface area (m^2^/g) on *q_t_*.

### 3.6 Dye Adsorption Kinetics

To determine the relative rate of each dye-MOF interaction, time-dependent adsorption measurements were collected. The adsorption profiles for Cur, MB, and MO reveal clear and systematic differences among the three samples (Figure 7). The adsorption kinetics were quantified by fitting the experimental *q_t_*-*t* data to the pseudo-first-order (PFO) and pseudo-second-order (PSO) models, enabling direct comparison of rate constants and goodness-of-fit across all dye–MOF combinations^43,44^ (Figure 8). The fitted PFO and PSO rate constants are summarized in Table 3, together with representative literature values for UiO-66-based adsorbents, to place the present results in context.

**Figure 7:**
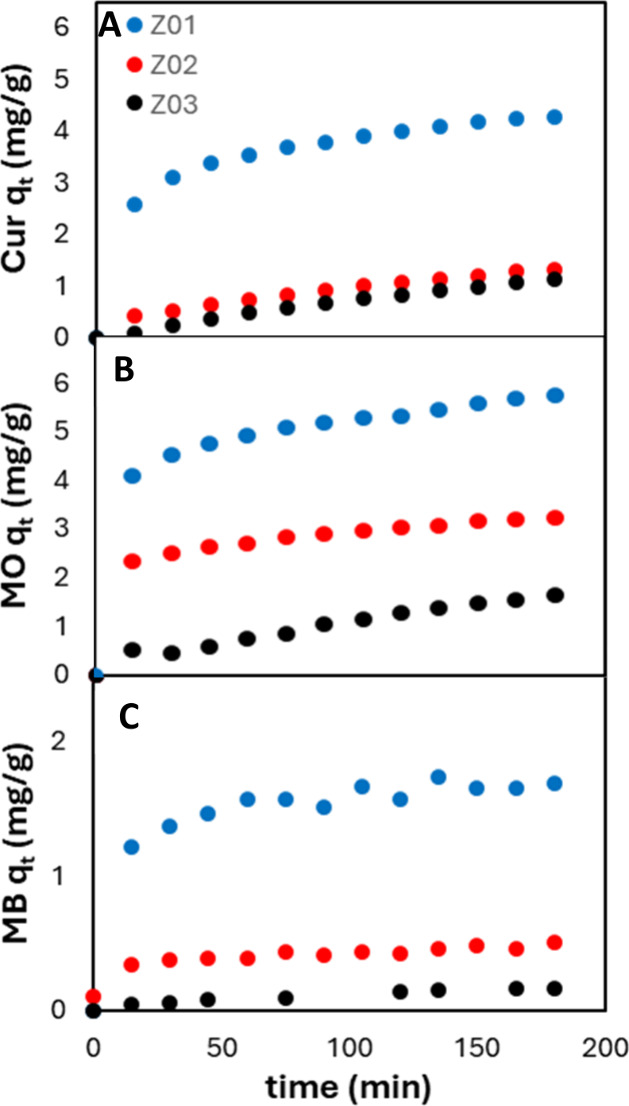
Time-dependent uptake profiles for curcumin (A), methyl orange (MO) (B), and methylene blue (MB) (C) by samples Z01 (blue), Z02 (red), and Z03 (black). Each graph displays adsorption behavior over 3 h.

**Table 3:** Comparison of Pseudo-First-Order (*k*_1_) and Pseudo-Second-Order (*k*_2_) Constants for MO, MB, and Curcumin Adsorption on UiO-66-Based Materials

	*k*_1_ (PFO)			*k*_2_ (PSO)			
	MO	Cur	MB	MO	Cur	MB	ref
Z01	1.14 × 10^–2^	1.39 × 10^–2^	1.19 × 10^–2^	1.59 × 10^–2^	1.18 × 10^–3^	7.00 × 10^–2^	this work
Z02	1.12 × 10^–2^	6.4 × 10^–3^	1.24 × 10^–2^	2.47 × 10^–2^	2.72 × 10^–3^	1.26 × 10^–1^	this work
Z03	3.0 × 10^–3^	2.0 × 10^–3^	4.6 × 10^–3^	1.33 × 10^–3^	1.98 × 10^–2^	4.32 × 10^–2^	this work
UiO-66(S)	7.47 × 10^–3^		7.66 × 10^–2^	1.64 × 10^–3^		8.4 × 10^–4^	^35^
UiO-66(U)	7.64 × 10^–3^		7.77 × 10^–2^	1.61 × 10^–3^		8.4 × 10^–4^	^35^
UiO-66			1.71 × 10^–2^			4.0 × 10^–4^	^26^
UiO-66					7.37 × 10^–4^		^28^
UiO-66-NH_2_					6.76 × 10^–4^		^28^
UiO-66-NO_2_	3.4 × 10^–2^		6.4 × 10^–2^	3.4 × 10^–2^		6.4 × 10^–2^	^45^

**Figure 8:**
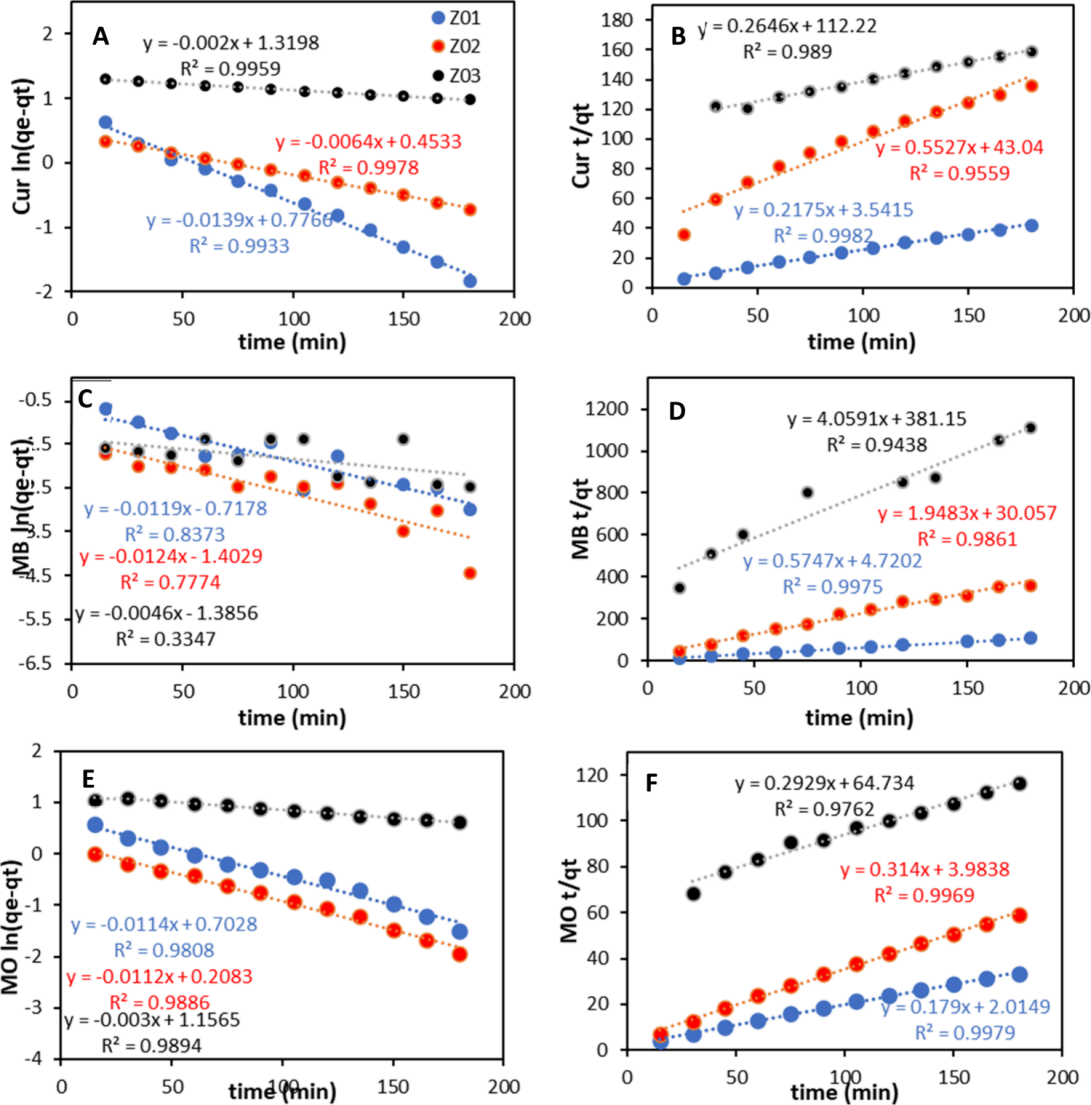
Kinetic modeling plots for curcumin (A, B), methylene blue (C, D), and methyl orange (E, F) adsorption by samples Z01 (blue), Z02 (red), and Z03 (black). Panels (A, C, E) depict pseudo-first-order fits (ln­(*q*_e_ – *q*_*t*_) vs time), while panels (B, D, F) show pseudo-second-order fits (*t*/*q_t_* vs time). Linear regression equations and *R* ^2^ values are included for each sample.

For Cur, both kinetic models describe the uptake behavior well, with PFO *R* ^2^ values of 0.9933 (Z01), 0.9978 (Z02), and 0.9959 (Z03), and PSO *R* ^2^ values of 0.9982 (Z01), 0.9559 (Z02), and 0.9890 (Z03). However, the PSO rate constants reveal a clear rate–capacity trade-off across the three materials. Z03 exhibits the highest PSO rate constant (*k*_2_ = 1.85 × 10^–2^ g·mg^–1^·min^–1^), indicating a faster apparent uptake under quiescent conditions, yet it reaches the lowest capacity at 180 min (*q*_180_) (∼1.13 mg/g). In contrast, Z01 shows the slowest PSO rate constant (1.18 × 10^–3^) but achieves the highest *q*_180_ (∼4.28 mg/g). Z02 remains intermediate in both rate and capacity. These results demonstrate that, for Cur, faster kinetic response does not translate into higher adsorption capacity, and the most effective material in terms of overall removal is Z01 despite its lower *k*_2_ value. This mixed behavior is broadly consistent with prior UiO-66 curcumin adsorption studies while the present results suggest that accessibility and transport constraints influence kinetic response.^28^

For MO, the time-dependent uptake is best described by the PSO model across all samples (*R* ^2^ = 0.9979, 0.9969, and 0.9762 for Z01, Z02, and Z03, respectively), although PFO fits remain reasonably high (*R* ^2^ ≈ 0.98–0.99), indicating mixed transport contributions under quiescent conditions.^35,45^ The PSO rate constants follow Z02 > Z01 ≫ Z03 [*k*_2_ = 2.47 × 10^–2^, 1.59 × 10^–2^, and 1.33 × 10^–3^ g·mg^–1^·min^–1^], suggesting that defect density accelerates initial uptake (Z02 has the highest defect level), while the very low *k*_2_ for Z03 is consistent with its low defect density and largest particle size. In contrast, the 180 min capacities rank Z01 > Z02 > Z03 [*q_t_* = 5.27, 3.04, 1.55 mg/g], showing that overall removal is governed primarily by accessibility: the smaller particles in Z01 shorten diffusion paths and expose more external surface even though Z02 is more defective.^19,39^ Taken together, and in light of MO’s anionic character, these results indicate that electrostatic attraction to Zr–oxo sites promotes rapid binding (reflected in higher PSO rates for the more defective Z02), whereas particle size controls the extent of uptake at longer times (giving Z01 the highest capacity).^25,46^ Thus, for MO, defects set the pace; size sets the ceiling.

Reported MB adsorption kinetics on UiO-66 vary across the literature. Pristine UiO-66 has, in some cases, been better described by the PFO model, whereas mesoporous or functionally modified UiO-66 materials more often exhibit stronger PSO behavior,^26,35,45^ suggesting that the apparent kinetic model depends on solution conditions, framework structure, and the fitting approach used. In this work, the kinetic data also clearly favor the PSO model, with *R* ^2^ values ranging from 0.9438 (Z03) to 0.9975 (Z01). Z02 exhibits the highest *k*_2_ (1.26 × 10^–1^ g·mg^–1^·min^–1^), followed by Z01 (6.997 × 10^–2^) and Z03 (4.323 × 10^–2^). These kinetic differences correspond to substantial differences in *q*_180_, with Z01 reaching ∼1.69 mg/g, Z02 ∼0.50 mg/g, and Z03 only ∼0.16 mg/g. Unlike curcumin, MB does not show rate–capacity inversion: materials with higher *k*_2_ values also achieve higher capacities, and Z01 and Z02 outperform Z03 in both respects.

The intraparticle diffusion (IPD) model was also applied to further probe the mass-transfer characteristics of curcumin adsorption on the Z-series materials.^44^ While the quiescent experimental conditions inherently limit the ability of the IPD model to unambiguously distinguish film diffusion from pore-diffusion contributions, the analysis remains valuable for identifying qualitative trends in transport behavior. In particular, the presence or absence of multilinearity, along with the magnitude of the intercepts, provides insight into whether adsorption proceeds through single- or multistep diffusion pathways and the relative importance of boundary-layer resistance across Z01, Z02, and Z03 (see Supporting Information)

Because of its superior performance, the adsorption mechanism of Z01 was further elucidated using the Langmuir and Freundlich models (Figure 9). The Freundlich model, which accounts for adsorption on heterogeneous surfaces and multilayer formation,^47^ yielded strong linear fits for all three compounds, with *R* ^2^ values of 0.9884 (Cur), 0.9523 (MO), and 0.9672 (MB). The Freundlich constants (*n*) derived from the slopes suggest favorable adsorption conditions, particularly for MO (*n* = 1.3483) and MB (*n* = 0.9065), while Cur (*n* = 0.3237) indicates a more gradual uptake profile.

**Figure 9:**
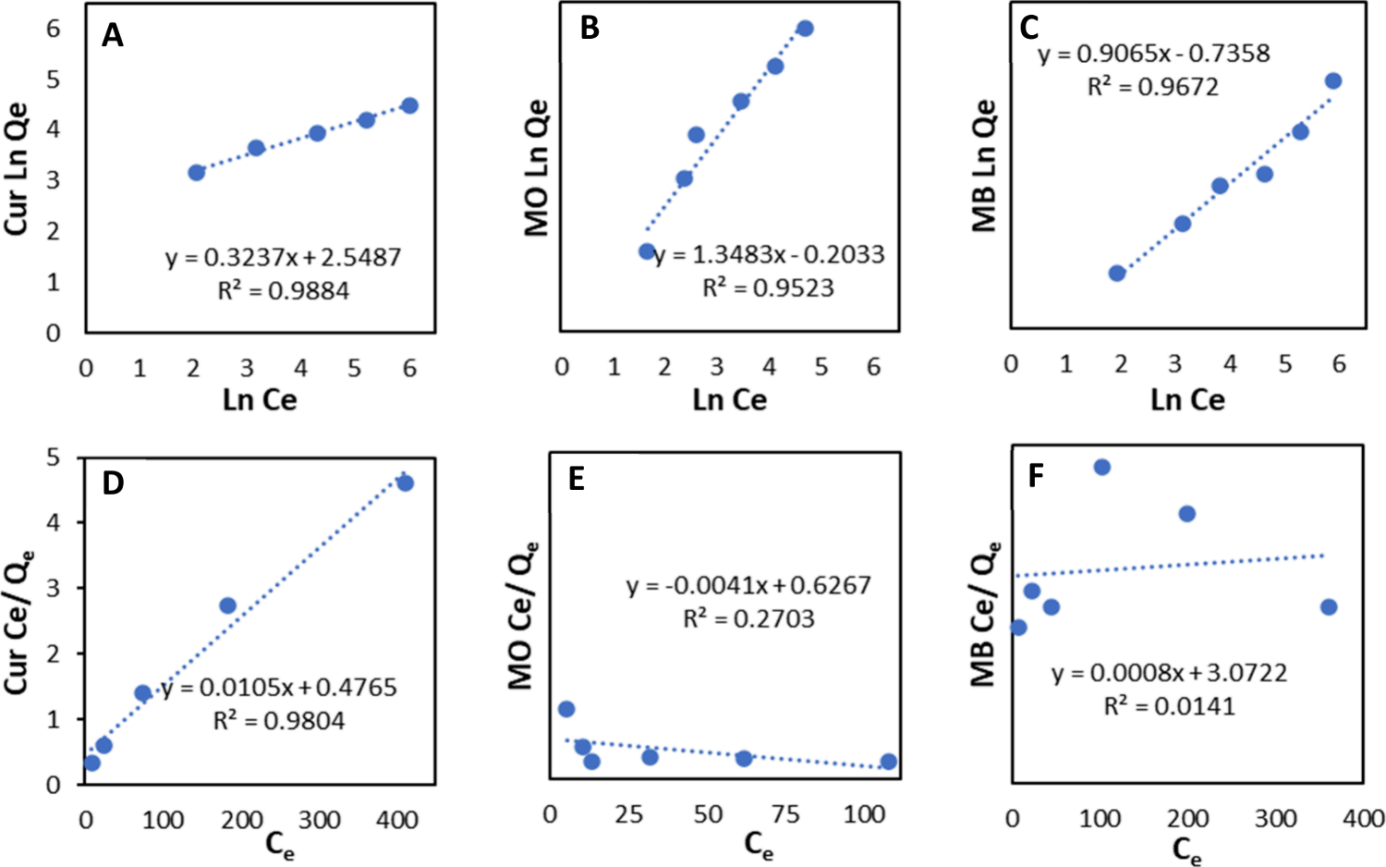
Adsorption isotherm modeling for curcumin (A, D), methyl orange (B, E), and methylene blue (C, F) on sample Z01. Panels (A–C) show Freundlich plots (ln *Q*_e_ vs ln *C*_e_), while panels (D–F) depict Langmuir plots (*C*_e_/*Q*_e_ vs *C*_e_). Linear regression equations and *R* ^2^ values are provided for each fit.

In contrast, the Langmuir model, which assumes monolayer adsorption on a homogeneous surface, showed a strong fit for Cur (*R* ^2^ = 0.9804), but poor correlation for MO (*R* ^2^ = 0.2703) and MB (*R* ^2^ = 0.0141). The linearity observed for Cur suggests that its adsorption onto Z01 may proceed via a well-defined monolayer mechanism with high site specificity. The negative slope in the MO Langmuir plot and the near-zero slope for MB indicate that these dyes do not conform to Langmuir assumptions, likely due to surface heterogeneity, electrostatic repulsion, or multilayer interactions.

Overall, the isotherm analysis reinforces the kinetic findings: Z01 exhibits strong and specific interactions with curcumin, consistent with both Langmuir and Freundlich models, while its performance with MO and MB is better described by the Freundlich model alone. These results underscore the importance of tailoring adsorption models to the physicochemical nature of the adsorbate and highlight Z01’s potential for selective uptake of neutral or moderately polar compounds.

Although postadsorption SEM/PXRD characterization was not performed in the present study, major morphological alteration of UiO-66 under the mild adsorption conditions used here is not expected based on the known stability of UiO-66 in aqueous and organic media.^22,46^ Therefore, the observed adsorption trends are interpreted primarily in terms of particle size, defect density, and accessibility rather than framework degradation. Direct postadsorption structural characterization will be addressed in future work.

## 4 Conclusions

This study shows that acetic-acid modulation provides a simple route to tune both particle size and defect density in UiO-66 and, importantly, to examine their distinct roles in dye adsorption. Increasing modulator concentration produced progressively larger particles, whereas defect density and BET surface area showed a nonmonotonic trend, with a maximum at intermediate acetic-acid loading. Extending the synthesis time from 6 to 48 h produced no measurable improvement in structure or adsorption performance, indicating that 6 h is sufficient under the present conditions.

Among the materials studied, Z01 showed the highest overall adsorption performance despite not having the highest defect density or surface area, indicating that, within the range examined, particle size and external accessibility influenced uptake more strongly than defect density alone. Adsorption was strongly adsorbate-dependent: methyl orange showed the highest uptake, methylene blue the lowest, and curcumin was more sensitive to diffusion limitations and accessibility. Consistent with earlier reports that synthesis conditions affect UiO-66 growth and defect formation, and that MOF adsorption depends strongly on adsorbate properties, the present work advances the field by distinguishing particle size and defect density as separate mechanistic variables across anionic, cationic, and neutral adsorbates within one UiO-66 series.

The study is limited to single-solute batch experiments under fixed conditions and without direct postadsorption structural characterization. Future work should therefore include postadsorption characterization of UiO-66, optimization of adsorption variables such as pH, temperature, adsorbent dose, and ionic strength, as well as reusability, multicomponent adsorption, and testing in real wastewater or other complex environmental matrices. Overall, these results provide a practical basis for designing UiO-66 adsorbents by jointly tuning accessibility and defect chemistry to match the target contaminant.

## Supplementary Material



## Notes

Table S1: Synthesis conditions for UiO-66 samples (Z01–Z24); Figure S1: Particle size distribution (DLS); Figure S2: Particle size evolution (SEM); Figure S3: BET surface area variation; Figure S4: Intraparticle diffusion (IPD) plots for dyes (curcumin, MB, and MO); Figure S5: Infrared (IR) spectra for samples Z01–Z03 (PDF)
