# Supplementary material for: Defect and Particle-Size Engineering as Mechanistic Drivers for Dye Uptake in a Zirconium Metal–Organic Framework
Source: ACS Omega. 2026 Jun 9;11(24):35389–99. doi: 10.1021/acsomega.6c00601 (PMC13294897; doi:10.1021/acsomega.6c00601)
Supplement: Supplementary file 1 [file ao6c00601_si_001.pdf]

Supporting Information

Defect and Particle-Size Engineering as Mechanistic Drivers for Dye Uptake in a Zirconium MOF

Karl Thomas Jackson*, Robert H. Lomax, Fatemeh Parnianchi

Department of Chemistry, College of Natural and Health Sciences, Virginia State University, Petersburg, VA, USA.

*Corresponding author's e-mail: kjackson@vsu.edu

List of Supporting Information

All entries follow SI-style ordering and formatting.

Table of Contents (Supporting Information)

- **Table S1.** Synthesis Conditions for UiO-66 Samples
- **Figure S1.** Particle size distribution by DLS
- **Figure S2.** Particle size evolution by SEM
- **Figure S3.** Surface area variation
- **Figure S4.** Intraparticle-diffusion (IPD) plots
- **Figure S5.** Infrared (IR) spectra

Table S1. Synthesis Conditions for UiO-66 Samples

Sample ID	Reaction Time (h)	Acetic Acid Equivalents
Z01	6	75 (1.7)
Z02	6	150 (3.4)
Z03	6	300 (5.2)
Z04	12	75 (1.7)
Z05	12	150 (3.4)
Z06	12	300 (5.2)
Z07	18	75 (1.7)
Z08	18	150 (3.4)
Z09	18	300 (5.2)
Z10	24	75 (1.7)
Z11	24	150 (3.4)
Z12	24	300 (5.2)
Z13	30	75 (1.7)
Z14	30	150 (3.4)
Z15	30	300 (5.2)
Z16	36	75 (1.7)
Z17	36	150 (3.4)
Z18	36	300 (5.2)
Z19	42	75 (1.7)
Z20	42	150 (3.4)
Z21	42	300 (5.2)
Z22	48	75 (1.7)
Z23	48	150 (3.4)
Z24	48	300 (5.2)

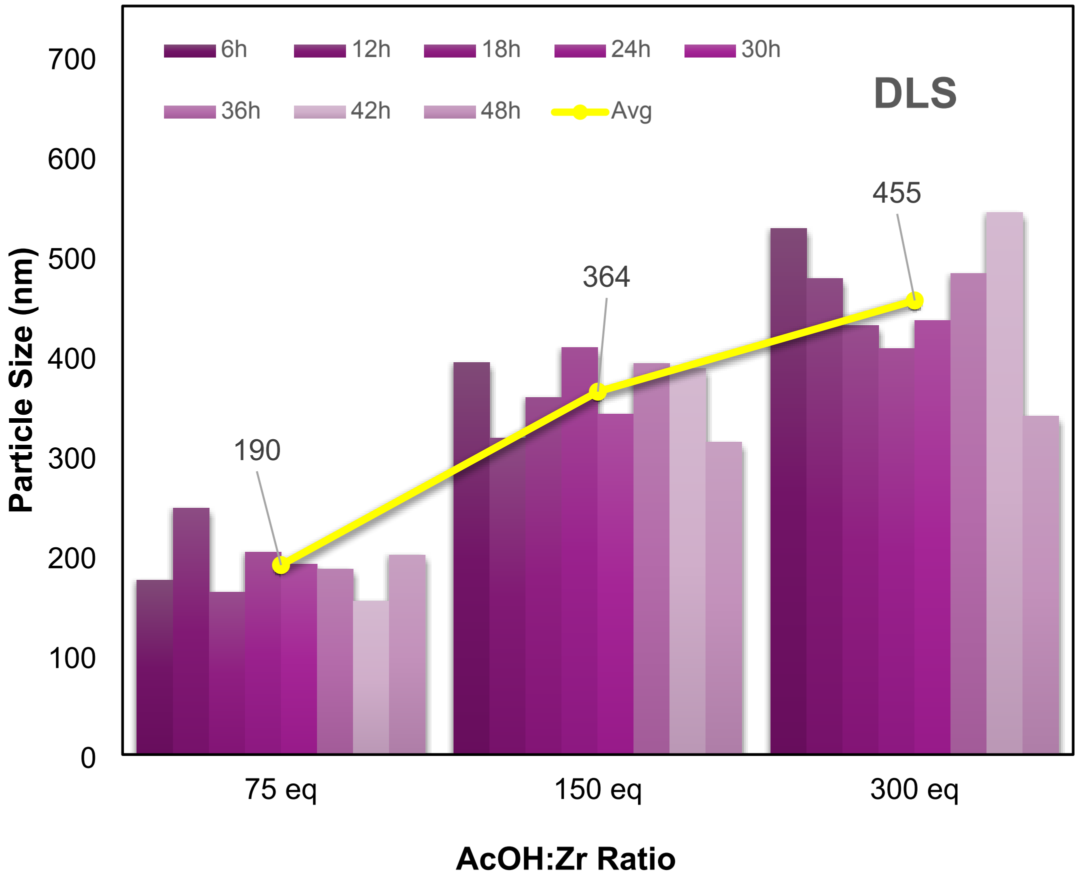

Figure S1. Particle size distribution of synthesized samples as a function of AcOH:Zr ratio and reaction time, determined via Dynamic Light Scattering (DLS). Bar plots represent particle sizes at time intervals ranging from 6 h to 48 h for AcOH:Zr ratios of 75, 150, and 300 equivalents. The yellow line traces the average particle size across each ratio, increasing from 190 nm (75 eq) to 364 nm (150 eq) and 455 nm (300 eq), indicating a positive correlation between modulator concentration and particle growth.

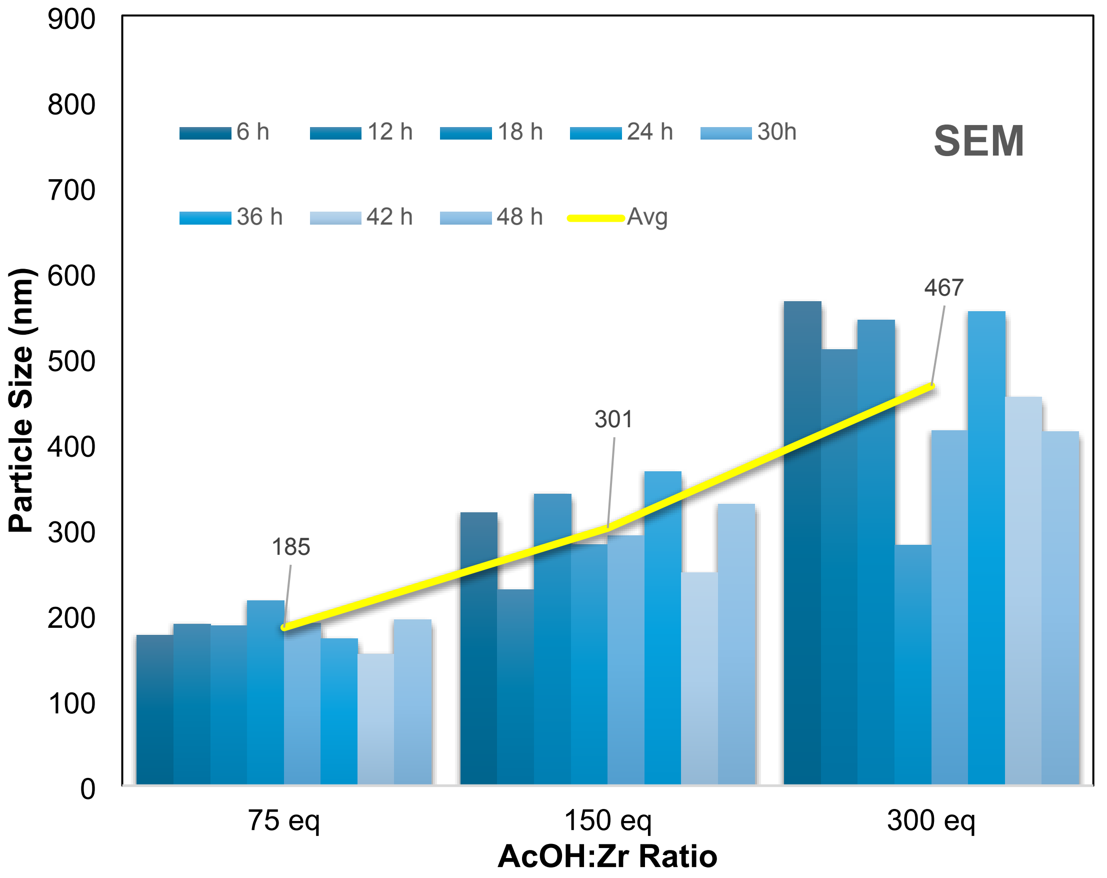

Figure S2. Particle size evolution of synthesized samples as a function of AcOH:Zr ratio and reaction time, measured via Scanning Electron Microscopy (SEM). Bar plots represent particle sizes at time intervals from 6 h to 48 h for AcOH:Zr ratios of 75, 150, and 300 equivalents. The yellow line indicates the average particle size at each ratio, increasing from 185 nm (75 eq) to 301 nm (150 eq) and 467 nm (300 eq), highlighting the modulator-dependent growth trend.

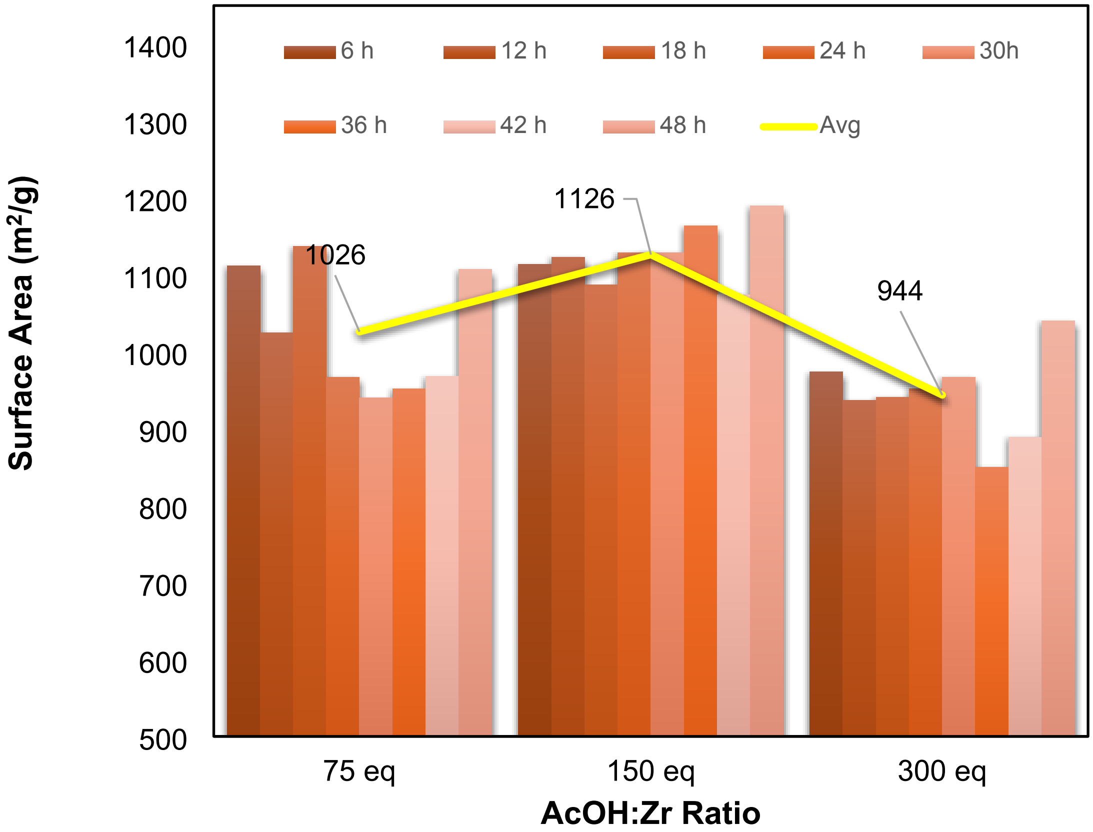

Figure S3. Surface area variation of synthesized samples as a function of AcOH:Zr ratio and reaction time. Bar plots represent BET surface areas measured at time intervals from 6 h to 48 h for AcOH:Zr ratios of 75, 150, and 300 equivalents. The yellow line traces the average surface area for each ratio, peaking at 1126 m^2/g for 150 eq, followed by 1026 m^2/g (75 eq) and 944 m^2/g (300 eq), suggesting an optimal modulator concentration for maximizing porosity.

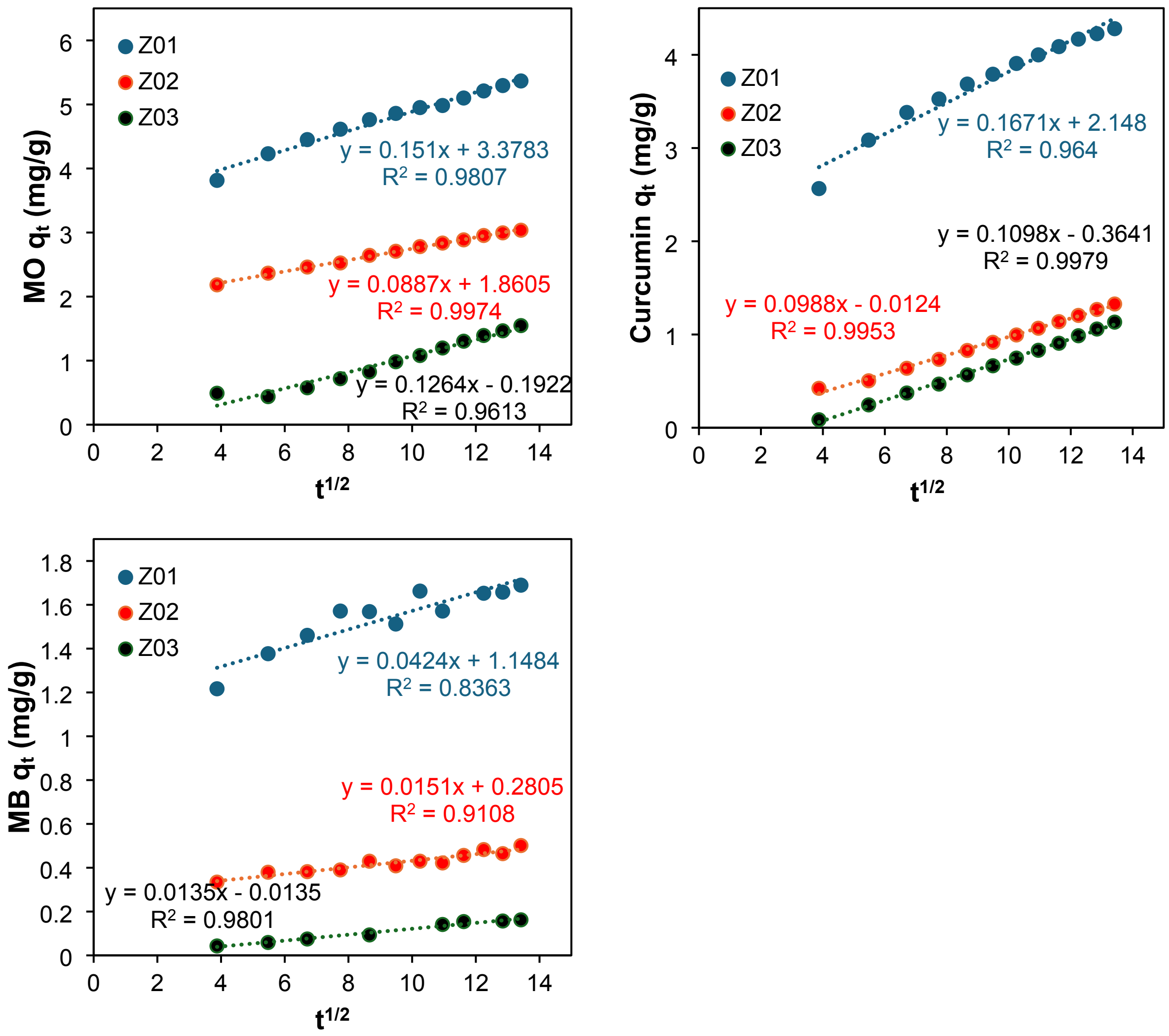

Figure S4.

Intraparticle-diffusion (IPD) plots for curcumin, MB, and MO on samples Z01–Z03. Curcumin shows strong linearity ($R^2 \approx 0.964$–0.998), indicating IPD-controlled kinetics. MB displays weaker linearity ($R^2 \approx 0.836$–0.980), suggesting additional kinetic pathways. MO maintains high linearity ($R^2 \approx 0.961$–0.997). Together, the profiles reflect adsorbate-dependent diffusion and boundary-layer effects.

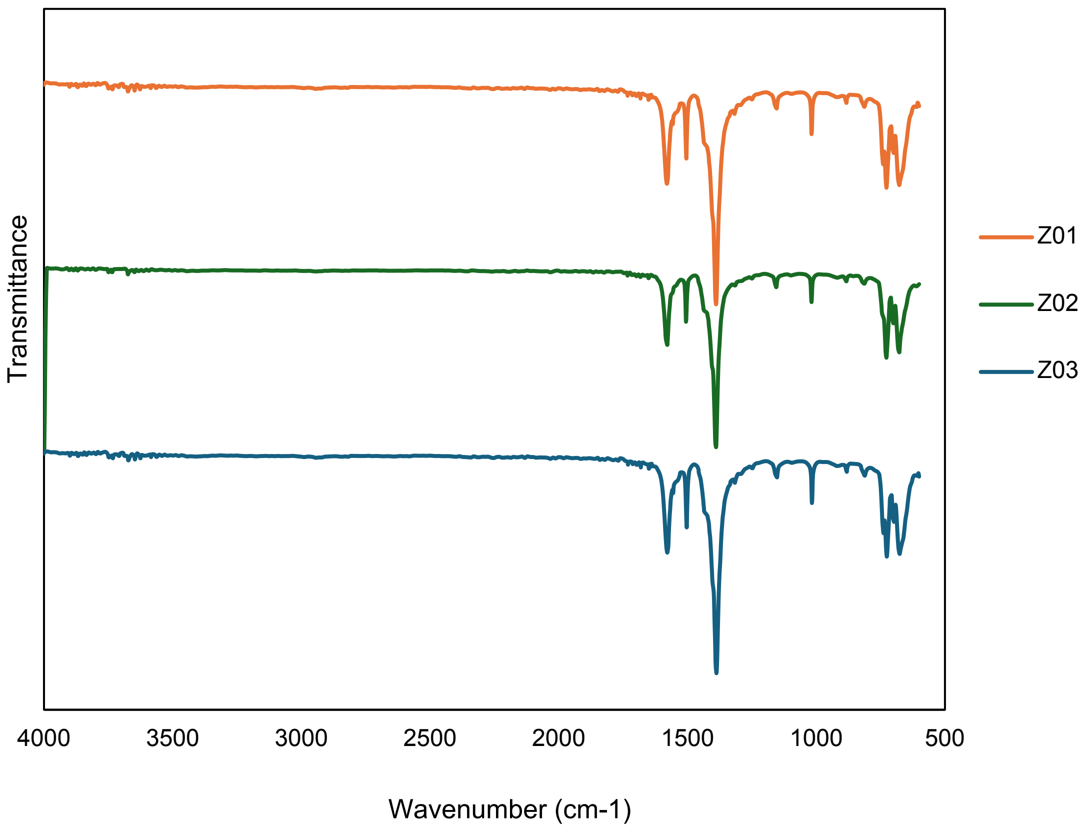

Figure S5. Infrared (IR) spectra of samples Z01 (orange), Z02 (green), and Z03 (blue), recorded over the range 4000–500 cm^{-1}.
